# Polystyrene-Al_2_O_3 _composite solid polymer electrolyte for lithium secondary battery

**DOI:** 10.1186/1556-276X-7-19

**Published:** 2012-01-05

**Authors:** Yu-Jeong Lim, Yu-Ha An, Nam-Ju Jo

**Affiliations:** 1 Department of Polymer Science and Engineering, Pusan National University, Jangjeon-dong, Geumjeong-gu, Busan, 609-735, South Korea

**Keywords:** polystyrene, Al_2_O_3_, solid polymer electrolyte, polymer-in-salt system, lithium secondary battery

## Abstract

In a common salt-in-polymer electrolyte, a polymer which has polar groups in the molecular chain is necessary because the polar groups dissolve lithium salt and coordinate cations. Based on the above point of view, polystyrene [PS] that has nonpolar groups is not suitable for the polymer matrix. However, in this PS-based composite polymer-in-salt system, the transport of cations is not by segmental motion but by ion-hopping through a lithium percolation path made of high content lithium salt. Moreover, Al_2_O_3 _can dissolve salt, instead of polar groups of polymer matrix, by the Lewis acid-base interactions between the surface group of Al_2_O_3 _and salt. Notably, the maximum enhancement of ionic conductivity is found in acidic Al_2_O_3 _compared with neutral and basic Al_2_O_3 _arising from the increase of free ion fraction by dissociation of salt. It was revealed that PS-Al_2_O_3 _composite solid polymer electrolyte containing 70 wt.% salt and 10 wt.% acidic Al_2_O_3 _showed the highest ionic conductivity of 9.78 × 10^-5 ^Scm^-1 ^at room temperature.

## Introduction

A lithium secondary battery using solid polymer electrolyte [SPE] is an attractive energy source for portable devices since the use of SPE makes the fabrication of safe batteries possible and permits the development of thin batteries with design flexibility. Most of the efforts to date have focused on poly(ethylene oxide) [PEO] as the host material for SPE [1-3]. However, it has a major drawback of having a low ionic conductivity (10^-8 ^to 10^-5 ^Scm^-1^) at room temperature [4]. Thus, many researchers [5-7] have focused on the SPE consisting of the polymer with low glass transition temperature [*T*_g_] and moderate concentrations of salt in order to overcome the low ionic conductivity of SPE, but high ambient conductivity has not yet been reached. Low ionic conductivity can be achieved from the fact that the ionic mobility strongly depends on the polymer segmental motion and that the cation transport number is low in the SPE at a high salt concentration. Therefore, new materials with unconventional conduction mechanisms are clearly needed [8].

In common SPEs, a polymer which has polar groups in the chain is necessary for electrolyte formation. The polar groups dissolve lithium salt and coordinate cations. The cations can move between coordinating sites in one chain or in neighboring chains, promoted by the segmental motion [9]. From this point of view, a polymer which has nonpolar groups is not suitable for the polymer matrix in common SPEs. However, in this new composite SPE consisting of polystyrene [PS] and having nonpolar groups, LiCF_3_SO_3 _and Al_2_O_3 _with polymer-in-salt system, the transport of cations is done by ion-hopping through an ion percolation path made of high content lithium salt instead of segmental motion. Moreover, Al_2_O_3 _can cause conductivity enhancement depending on the nature of the filler surface group [10]. In this work, the ionic conductivity of PS-Al_2_O_3 _composite SPE according to the salt content was checked, and the effect of Al_2_O_3 _type and content on ion conduction properties in PS-based composite SPE was investigated.

## Experimental section

### Materials

Polystyrene (Sigma-Aldrich Corporation, St. Louis, MO, USA) with a number average molecular weight (*M_n_*) of 170,000 was used as received without undergoing further purification process. As salt, LiCF_3_SO_3 _(Sigma-Aldrich Corporation) was dried and stored in a desiccator under nitrogen. Three types of aluminum oxides [Al_2_O_3_] (Sigma-Aldrich Corporation) with acidic, neutral, and basic surface groups as fillers were also used. As an organic solvent, *N*-butyl acetate (Junsei Chemical Co., Ltd., Chuo-ku, Tokyo, Japan) was used in order to dissolve the materials.

### Preparation of PS-based composite SPE films

An appropriate amount of PS was introduced into *N*-butyl acetate and stirred for 24 h; after that, a definitive amount of LiCF_3_SO_3 _was added to the solution and stirred again for 24 h. At the same time of the PS/LiCF_3_SO_3 _solution preparation, 5, 10, 15, and 20 wt.% Al_2_O_3 _were added to *N*-butyl acetate. Then, the solution was sonicated for 10 min and stirred for 24 h for dispersion. PS-based SPE was prepared by mixing the PS/LiCF_3_SO_3 _and Al_2_O_3 _solutions for 4 days. The solutions were directly cast on 3 × 3 cm^2 ^stainless steel plates after mixing and then allowed to dry in a vacuum oven for 5 days at 40°C.

### Characterization

Ionic conductivity of the sample was measured by Gamry Instruments' (Warminster, PA, USA) Reference 600 impedance analyzer. Deconvolution of the composite bands of the Fourier transform infrared [FT-IR] spectra was accomplished by the best fits of constituent Gaussian peaks, and the fractions of salt forms were calculated by the peak fitting program of Origin 7.0 software (OriginLab Corporation, Northampton, MA, USA) to analyze the change of salt forms in SPEs. Scanning electron microscopy [SEM] was also used to observe the morphology of the specimen.

## Results and discussion

### Ionic conductivity

#### Ionic conductivities of PS-based composite SPEs with salt content

**Figure 1:**
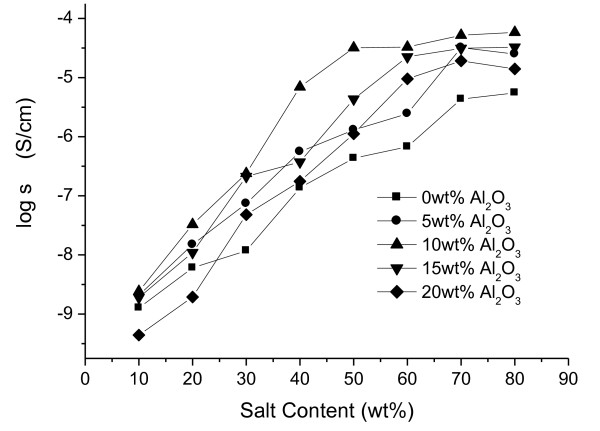
**Ionic conductivities of PS-based composite SPEs**. PS-based composite SPEs with 0, 5, 10, 15, and 20 wt.% Al_2_O_3 _and various salt contents.

Figure 1 shows the ionic conductivities of PS-based SPEs with 0, 5, 10, 15, and 20 wt.% Al_2_O_3 _and various salt contents. In the common SPEs, the ionic conductivity increased with the salt content up to its peak and then the ionic conductivity decreased because the polymer mobility decreased and *T*_g _of the polymer increased as the salt content increased. However in PS-based composite SPEs, there is no decrease in the ionic conductivity because PS does not contribute to the dissociation of salts and transport of cations. The transport of cations is done by ion-hopping through the ion percolation path made of high content of salt instead of segmental motion, so the ionic conductivities increased with salt content.

Moreover, Al_2_O_3 _can dissolve the lithium salt instead of the polar groups in the polymer matrix using Lewis acid-base interactions between the surface group of Al_2_O_3 _and salt [10]. The sample consisting 70 wt.% salt and 10 wt.% Al_2_O_3 _shows the highest ionic conductivity of 5.83 × 10^-5 ^Scm^-1^.

#### Ionic conductivity of PS-based composite SPE according to Al_2_O_3 _type and content

**Figure 2:**
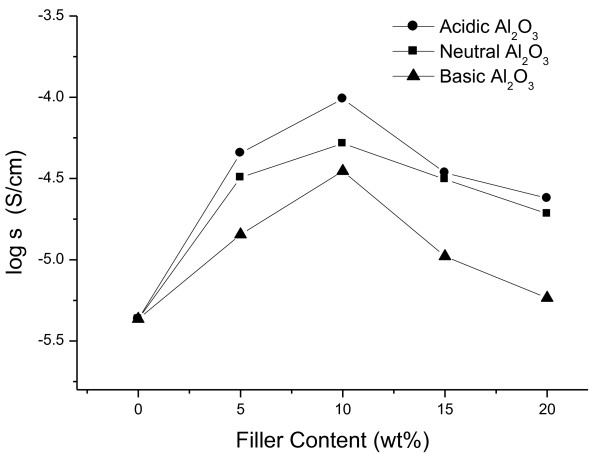
**Ionic conductivities of PS-based composite SPEs with different types of Al_2_O_3_**.

Figure 2 shows the ionic conductivities of PS-based composite SPEs consisting 70 wt.% salt and different types of Al_2_O_3_. The ionic conductivity increased up to 10 wt.% Al_2_O_3 _and then decreased, irrespective of the Al_2_O_3 _type. As the content of Al_2_O_3 _increased over 10 wt.%, the aggregates of Al_2_O_3 _was observed in all types of Al_2_O_3_. This is related to the decrease in ionic conductivity at above 10 wt.% Al_2_O_3_. Among the samples, the maximum ionic conductivity was found for SPE with acidic Al_2_O_3_, and the ionic conductivity decreased in the order of SPEs with acidic, neutral, and basic Al_2_O_3_. This tendency may be related to the number of free ions by dissociation of salt. Salt can be dissociated by the interaction between salt anions and surface OH groups of Al_2_O_3_. Acidic Al_2_O_3 _has had the most OH groups which interact with salt, so the SPE having acidic Al_2_O_3 _can have the highest free-ion numbers. Neutral Al_2_O_3 _has had the second amount of surface OH groups, and basic Al_2_O_3 _has had the least surface OH groups. Thus, the ionic conductivity decreased in that order, and the highest ionic conductivity of 9.78 × 10^-5 ^Scm^-1 ^could be obtained at 10 wt.% acidic Al_2_O_3_.

### FT-IR evidence of dissociated ions

In the polymer-in-salt system, ion clouds made of ion aggregates and ion pairs play an important role in ion conduction [11], so it is necessary to investigate the change of salt form of the SPE films as salt concentrations increase. FT-IR spectra have confirmed the presence of ion pairs and aggregates in SPEs based upon poly(propylene oxide) [12-16] and PEO [17-24]. A clear distinction between free (dissociated) ions, contact ion pairs, and more aggregates may be observed in the vibrational spectra of the internal mode of anions, such as the triflate anion [Tf^-^]. Ion association occurs at the SO_3 _end of the anion; thus, the symmetric SO_3 _stretching mode is highly sensitive to change in the coordination state of the anion. Band fitting of these regions has provided information pertaining to the types of aggregation and strengths of ionic interactions occurring in the SPEs.

Above a certain salt concentration, the symmetric SO_3 _stretching mode is found to consist of two or more peaks. The different anion environments may be attributed to ion association, in consideration of the nondegenerate A_1 _symmetry of this mode. The symmetry of an anion is lowered by coordination to a cation. Band fitting of the SO_3 _regions reveals the peak components which arise from various ion aggregates. Higher frequency components, corresponding to higher aggregates, may be observed with further increase in salt concentration [25]. Assignments for bands observed in the symmetric SO_3 _stretching regions are summarized in Table 1[18,26].

**Table 1:** Some band assignments for triflate species

Band	Wave number (cm^-1^)	Assignment
*ν*_s_(SO_3_)	1,032	Free Tf^- ^ions, solvent-separated pairs
	1,040	Ion pairs (LiTf), LiTf_2_^-^, LiTf_3_^2-^
	1,051	Li_2_Tf^+ ^aggregate
	1,062	Li_3_Tf^2+ ^aggregate

**Figure 3:**
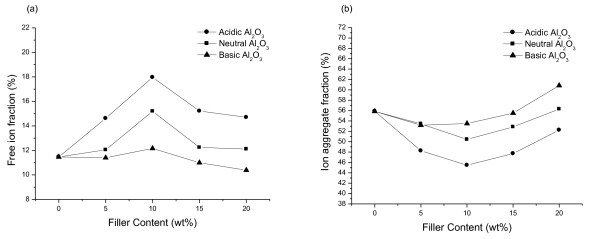
**Fraction types of PS-based composite SPEs**. (**a**) Free-ion fractions and (**b**) ion aggregate fractions of PS-based composite SPEs having 70 wt.% salt with various types of Al_2_O_3_.

When Al_2_O_3 _is added, the anions have greater affinity toward the Al_2_O_3 _surface acid groups than the cations. Due to the polarizability of the Tf^-^, a strong affinity can be expected between the Tf^- ^and the Al_2_O_3 _surface acid groups. It results in the dissociation of the salt and makes the cations free [10,27]. The free-ion and ion aggregate fractions of PS-based composite SPEs having 70 wt.% salt with various types of Al_2_O_3 _are shown in Figure 3. As shown in Figure 3, for all cases, the free-ion fractions of SPE consisting 70 wt.% salt increased until the content of Al_2_O_3 _reached 10 wt.%. By adding more Al_2_O_3_, the free-ion fraction decreased. SPE with acidic Al_2_O_3 _having the most OH groups which interact with salt had the highest free-ion fraction. The free-ion fraction decreased in the order of SPEs with acidic, neutral, and basic Al_2_O_3_. This tendency was similar to that of the ionic conductivity. From this result, it could be known that the ionic conductivity was mainly influenced by the free-ion fraction. Also, the highest ionic conductivity could be obtained in the case of SPE with 10 wt.% acidic Al_2_O_3 _whose free-ion fraction was the highest and ion aggregate fraction was the lowest.

### SEM images

**Figure 4:**
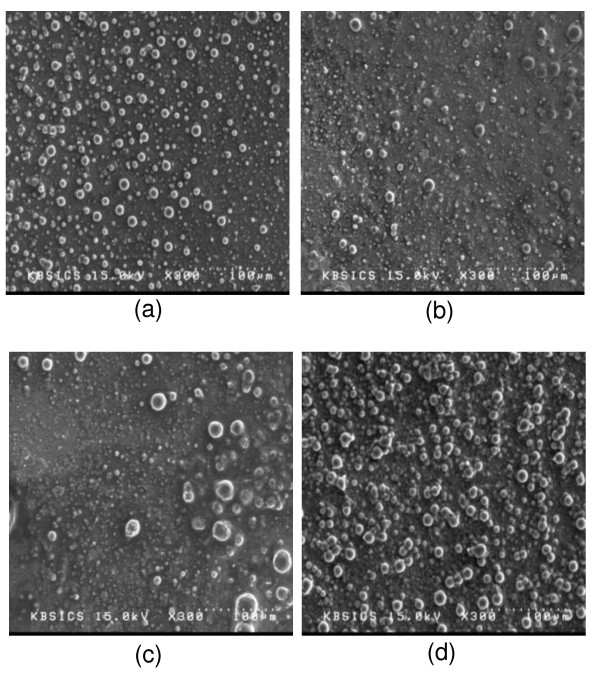
**SEM images of PS-based composite SPEs**. PS-based composite SPEs with (**a**) 5 wt.%, (**b**) 10 wt.%, (**c**) 15 wt.%, and (**d**) 20 wt.% Al_2_O_3 _contents at a magnification of ×300.

Figure 4 shows the SEM images of PS-based composite SPEs with Al_2_O_3 _content. From the SEM images, we can see that the fillers are well dispersed, and there are no aggregates of Al_2_O_3 _until 10 wt.% Al_2_O_3 _is reached, but as more Al_2_O_3 _was added, the aggregates of fillers which might disturb the ion transport were observed. Thus, the ionic conductivity of PS-based composite SPEs, as shown in Figure 2, increased up to 10 wt.% Al_2_O_3_, and then the ionic conductivity decreased.

## Conclusions

Composite SPEs based on PS, LiCF_3_SO_3_, and Al_2_O_3 _were prepared, and the effect of the Al_2_O_3 _type and content on ion conduction properties of SPEs was investigated. As the salt content increased, the ionic conductivities increased continuously. In contrary of the common SPE, PS-based composite SPE has no decrease in ionic conductivity because PS does not contribute to the dissociation of salts and transport of cations. As the Al_2_O_3 _content increased, the ionic conductivity increased until the content of Al_2_O_3 _reached 10 wt.%. Then, the sample consisting 70 wt.% salt and 10 wt.% Al_2_O_3 _shows the highest ionic conductivity of 5.83 × 10^-5 ^Scm^-1^. The maximum ionic conductivity was found in SPE with acidic Al_2_O_3_. The ionic conductivity decreased in the order of SPEs with acidic, neutral, and basic Al_2_O_3_. This tendency may be related to the number of free ions by dissociation of salt. The SEM images show that the fillers are well dispersed, and there is no aggregate of fillers until 10 wt.% Al_2_O_3 _is reached. On the other hand, as more Al_2_O_3 _contents were added, the aggregates of fillers appeared. It seems that the aggregates of fillers disturb the ion transport, so the ionic conductivity increased up to 10 wt.% Al_2_O_3_, and then the ionic conductivity decreased. The highest ionic conductivity of 9.78 × 10^-5 ^Scm^-1 ^could be obtained at 10 wt.% acidic Al_2_O_3_.

## Competing interests

The authors declare that they have no competing interests.

## Authors' contributions

YJL and YHA carried out the measurements and analysis of data. NJJ commented on various points and drafted the manuscript. All authors read and approved the final manuscript.
